# Patterns and Predictors of Self‐Regulatory Fatigue Among Intensive Care Unit Nurses: A Person‐Centered Latent Profile Analysis

**DOI:** 10.1155/jonm/4685274

**Published:** 2026-10-06

**Authors:** Hongli Zhang, Yilin Wang, Mengyi Hu, Yanting Han, Jiao Zhou, Qiaoying Yang, Ka Li

**Affiliations:** ^1^ West China School of Nursing Sichuan University/West China Hospital Sichuan University Chengdu China scu.edu.cn; ^2^ Medicine and Engineering Interdisciplinary Research Laboratory of Nursing & Materials West China Hospital Sichuan University/West China School of Nursing Sichuan University Chengdu China scu.edu.cn; ^3^ Xi’an Weiyang Baoshihua Xinglong Yuan Hospital Xi’an Shaanxi China; ^4^ Xiong’an Xuanwu Hospital, No. 1 Xili Road Xiong’an New Area, Baoding, Hebei, China

**Keywords:** ICU nurses, latent profile analysis, self-regulatory fatigue, surface acting, work engagement

## Abstract

**Background:**

Intensive care units (ICUs) play a critical role in the public health emergency response system. ICU nurses are the backbone of critical care and must continuously engage in self‐regulation while working in highly demanding environments, which may lead to self‐regulatory fatigue. Self‐regulatory fatigue may affect work engagement and patient safety. However, little is known about the profiles of self‐regulatory fatigue among ICU nurses and the factors associated with profile membership.

**Objective:**

To identify profiles of self‐regulatory fatigue among ICU nurses and examine factors associated with profile membership.

**Methods:**

A cross‐sectional survey was conducted between February and August 2025. A total of 687 ICU nurses completed an online questionnaire that collected general demographic information and assessed self‐regulatory fatigue, surface acting, and work engagement. Differences among latent profiles were examined using descriptive statistics, univariable analyses, and logistic regression analyses.

**Results:**

Three latent profiles were identified: “Low self‐regulatory fatigue (Class 1),” “Moderate self‐regulatory fatigue (Class 2),” and “High self‐regulatory fatigue (Class 3),” accounting for 32.3%, 52.7%, and 15.0%, respectively. In multivariable logistic regression analyses, lower work engagement was independently associated with membership in the moderate self‐regulatory fatigue profile compared with the low self‐regulatory fatigue profile. However, work engagement was not independently associated with membership in the high self‐regulatory fatigue profile compared with the low self‐regulatory fatigue profile. Associations of demographic and work‐related factors with profile membership differed across the comparisons.

**Conclusion:**

The level of self‐regulatory fatigue among ICU nurses showed a heterogeneous distribution, with over two‐thirds of the participants classified into the moderate or high self‐regulatory fatigue profiles. Identifying the sociodemographic and work‐related predictors associated with self‐regulatory fatigue profiles can facilitate the implementation of tailored interventions for specific subgroups and improve their effectiveness.

**Implications for Nursing Management:**

The findings may help nursing managers understand the heterogeneous profiles of self‐regulatory fatigue among ICU nurses. Identifying different profiles of self‐regulatory fatigue may inform targeted strategies to alleviate such fatigue and promote continuous quality improvement.

## 1. Introduction

The intensive care unit (ICU) is a specialized hospital unit that provides care for patients with life‐threatening conditions and serves as a cornerstone of the public health emergency response system [1]. As key members of the critical care workforce, ICU nurses are responsible for implementing treatment plans, dynamically monitoring changes in patients’ conditions, and delivering emergency medical interventions. Their work reflects the capacity of modern healthcare to treat critically ill patients and embodies advanced clinical nursing practice [2, 3]. ICU nurses are required to possess keen clinical observation skills and the ability to rapidly provide life‐support interventions in critical situations. They must also provide emotional support to unaccompanied patients and proactively respond to family members’ concerns and emotional needs. ICU nurses are frequently exposed to moral dilemmas, including patient suffering, death, and requests for futile care [4, 5]. They work continuously in environments characterized by severe patient conditions, technical complexity, and high emotional intensity [6]. Consequently, ICU nurses must maintain sustained attention, emotional stability, and standardized behaviors in their work. Under high‐risk, high‐workload, and emotionally demanding conditions, they must continuously adjust their thoughts and behaviors to meet job demands. Such prolonged and persistent self‐regulatory efforts may deplete individuals’ limited psychological resources and ultimately lead to self‐regulatory fatigue.

Self‐regulation refers to the process of regulating one’s cognitive, emotional, and behavioral processes, including inhibiting dominant responses [7]. Its specific manifestations include cognitive regulation, such as controlling impulses, setting goals, and making decisions; emotional regulation, which involves managing emotional responses; and behavioral regulation, which refers to actively monitoring and adjusting one’s behavior to adapt to specific situations and demands [8]. Self‐regulatory ability is crucial for nurses’ physical and mental health, patient safety, and nursing quality [9]. According to the limited‐resource theory of self‐control, self‐regulatory efforts consume individuals’ limited psychological resources. Prolonged or continuous self‐regulation may impair self‐control capacity and lead to self‐regulatory fatigue [10].

Self‐regulatory fatigue refers to a range of complex cognitive, emotional, and behavioral problems resulting from the depletion of psychological resources after prolonged emotional management, sustained cognitive effort, and behavioral restraint [11]. Over the past decade, self‐regulatory fatigue has attracted considerable scholarly attention and prompted extensive empirical research. Studies have shown that self‐regulatory fatigue negatively affects decision‐making, coping, self‐management, and task performance, and may increase negative emotions, such as anxiety and depression [12–14]. It is also closely associated with burnout, impaired nurse–patient relationships, compromised patient safety, and high nurse turnover rates [15–17]. A study conducted during the COVID‐19 pandemic found that demanding work conditions, including closed‐loop management, restrictions on family accompaniment, expanded nursing responsibilities, prolonged exposure to critically ill patients and death, and frequent contact with anxious patients and family members, contributed to nurses’ self‐regulatory fatigue, work alienation, and mental health problems [18]. These challenging job demands are particularly prominent in ICUs [19]. Insufficient staffing, heavy workloads, the need to maintain a high level of alertness, and confined working environments may undermine nurses’ self‐regulatory capacity and ultimately trigger self‐regulatory fatigue. Existing studies on self‐regulatory fatigue have primarily focused on pediatric nurses [20], operating room nurses [16], and oncology nurses [21], whereas relatively little is known about this phenomenon among ICU nurses.

With the development of nursing disciplines and clinical practice, evaluation indicators of nursing quality now cover not only professional knowledge and practical skills but also humanistic care, including understanding patients, communicating effectively with them, and providing psychological and emotional support [22, 23]. This requires nurses to engage in emotional labor in addition to physical and intellectual work. In clinical nursing practice, nurses provide emotional support and convey empathy to patients through facial expressions, words, and actions [24]. Emotional labor is generally divided into surface acting and deep acting. Surface acting is a form of emotional concealment in which nurses deliberately disguise or suppress their true emotions to meet the emotional expectations of their work roles. In contrast, deep acting involves modifying one’s inner feelings to genuinely express the emotions required by the work role [25]. Studies have shown that surface acting is positively associated with stress, job dissatisfaction, emotional exhaustion, burnout, and turnover intention, and negatively associated with work engagement [26–28]. Persistent discrepancies between expressed emotions and inner feelings may undermine trust and communication with patients, thereby contributing to self‐regulatory fatigue [29]. ICU settings often impose restrictions on family accompaniment, which may lead to heightened uncertainty and emotional distress among patients and their family members. ICU nurses therefore need to provide patients and family members with continuous, timely, and individualized emotional support. Furthermore, when communicating with patients and family members about a patient’s condition, treatment decisions, and prognosis, ICU nurses must combine emotional support with clear explanations of medical information and emotional guidance, while helping to stabilize family members’ emotions [30, 31]. These specific demands expose ICU nurses to intense emotional labor and may result in patterns of self‐regulatory fatigue that differ from those observed among ward‐based nursing staff.

Work engagement is defined as a positive, fulfilling, work‐related psychological state [32]. It comprises three core dimensions: vigor, dedication, and absorption. Vigor refers to high levels of energy and mental resilience at work, willingness to invest effort in one’s work, and persistence in the face of difficulties. Dedication refers to a strong sense of significance, enthusiasm, responsibility, and accomplishment at work. Absorption refers to being fully concentrated on and happily immersed in one’s work [33]. Studies have shown that work engagement is positively associated with better patient outcomes, job satisfaction, nursing performance, and occupational well‐being, and negatively associated with burnout, self‐regulatory fatigue, and turnover intention [34–36]. Given the complexity of treatment regimens in ICUs and the vulnerability of critically ill patients, nursing care and clinical decisions may directly affect patient safety. Maintaining work engagement under the highly demanding conditions of the ICU presents a major challenge for ICU nurses. Nurses experiencing prolonged self‐regulatory fatigue may struggle to sustain the vigor, dedication, and absorption required for their duties, which may in turn impair work engagement and undermine job motivation, enthusiasm, and the perceived meaningfulness of their roles.

Critical care nursing is an essential component of modern healthcare, and ICU nurses play a central role in caring for critically ill patients [37]. Reducing self‐regulatory fatigue among ICU nurses and examining its profiles and associated factors are important for improving the quality of intensive care. To provide a theoretical basis for the present study, we draw on Goffman’s dramaturgical theory [38] and the conservation of resources (COR) theory [39] to explain how emotional labor demands and resource depletion may contribute to self‐regulatory fatigue. Specifically, Goffman’s dramaturgical theory provides insight into surface acting, emotional expression, and impression management in occupational settings, whereas COR theory elucidates the mechanisms through which ongoing resource depletion leads to psychological exhaustion.

Dramaturgical theory conceptualizes social interaction as a form of performance in which individuals manage the impressions they convey to others. Individuals engage in impression management to shape others’ perceptions and fulfill socially expected roles. ICUs are often characterized by restricted family accompaniment, frequent multidisciplinary collaboration, emergency situations, and complex interpersonal communication. These work demands may require ICU nurses to suppress their genuine emotions and display emotions that are consistent with professional norms. To maintain a professional and credible image, nurses may engage in surface acting when communicating with patients, family members, and other healthcare professionals. Sustained impression management may deplete individuals’ limited psychological resources over time. Such psychological resource depletion may impair self‐regulatory capacity, reduce cognitive and behavioral performance, and ultimately contribute to self‐regulatory fatigue.

COR theory posits that individuals strive to obtain, retain, foster, and protect resources that they value. These encompass personal, social, material, and energetic resources. Specifically, in intensive care contexts, such resources primarily involve psychological capital (e.g., resilience and self‐efficacy), emotional stability, and social support. When resource loss exceeds resource replenishment, individuals may experience maladaptive psychological states [40, 41]. From the perspective of COR theory, surface acting may function as a resource‐depleting work demand, whereas work engagement, characterized by vigor, dedication, and absorption, may help individuals cope with work demands and mitigate the adverse effects of resource loss. When surface acting continuously drains personal resources, and work engagement is insufficient to offset this loss, nurses may experience diminished cognitive control and impaired emotional and behavioral regulation, which may contribute to varying levels of self‐regulatory fatigue. Accordingly, we propose that surface acting among ICU nurses may be associated with the depletion of emotional and psychological resources during emotional regulation at work, thereby exacerbating self‐regulatory fatigue. Continuous resource loss may also hinder nurses’ ability to maintain effective self‐regulation, vigor, dedication, and absorption at work.

Evans et al. found that individuals differ in their capacity for self‐regulation and that self‐regulatory failure may occur in some individuals but not in others under similar circumstances [42]. Some studies have reported moderate levels of self‐regulatory fatigue among nurses [43], whereas others have reported high levels [44]. Previous studies have primarily described self‐regulatory fatigue among nurses at the aggregate level, without adequately considering the heterogeneity of its cognitive, emotional, and behavioral manifestations. This lack of attention to heterogeneity may limit the development of effective interventions for self‐regulatory fatigue, resulting in narrowly targeted populations and settings, one‐dimensional intervention designs, and limited reproducibility in implementation [45–48]. Identifying such heterogeneity may not only help clarify distinct patterns of psychological resource depletion theoretically but also provide evidence for developing tailored intervention strategies that correspond to the fatigue characteristics of different nursing subgroups in clinical practice.

Latent profile analysis (LPA) is a person‐centered statistical approach that identifies unobserved subgroups of individuals with similar response patterns across continuous observed variables [49]. Examining self‐regulatory fatigue only at the overall group level may obscure meaningful heterogeneity among nurses. By identifying distinct profiles of self‐regulatory fatigue among ICU nurses and examining factors associated with profile membership, LPA may provide a more nuanced and interpretable understanding of self‐regulatory fatigue than traditional variable‐centered approaches. Such findings may inform targeted interventions to reduce self‐regulatory fatigue among ICU nurses.

Accordingly, this study proposes three hypotheses: (1) distinct profiles of self‐regulatory fatigue exist among ICU nurses; (2) sociodemographic characteristics are associated with profile membership; and (3) surface acting and work engagement further predict such membership, with their effects differing across subgroups.

## 2. Methods

### 2.1. Study Design

This multicenter, cross‐sectional descriptive study was conducted to investigate self‐regulatory fatigue and its associated factors among ICU nurses from seven provinces (Shandong, Shaanxi, Hebei, Jiangsu, Sichuan, Hunan, and Liaoning) and two municipalities (Beijing and Chongqing) in China. Data were collected using a self‐administered online questionnaire that included items assessing participants’ sociodemographic characteristics, surface acting, self‐regulatory fatigue, and work engagement. We followed the guidelines recommended in the Strengthening the Reporting of Observational Studies in Epidemiology (STROBE) statement [50].

### 2.2. Participants

From February to August 2025, participants were recruited using convenience sampling. Hospital selection considered accessibility, the feasibility of study implementation, and the diversity in sampling sources. Efforts were made to recruit participants from ICUs with broadly comparable clinical functions across participating hospitals. A total of 687 ICU nurses were recruited from ICUs in tertiary hospitals across nine provinces and municipalities in China.

Before distributing the questionnaire, we contacted the administrative staff at each participating hospital to obtain permission to conduct the survey. Head nurses in the participating ICUs assisted with distributing the online questionnaire through WeChat, a widely used social media platform in China. The first page of the questionnaire provided information on the study purpose, significance, participation procedures, and informed consent. Participants could access the survey only after providing electronic informed consent. All participants were informed that participation was voluntary and that they could withdraw from the study at any time without penalty. Eligible participants were registered nurses who had worked in an ICU for at least 1 year. Nurses undergoing advanced training, nursing interns, and nurses who were unavailable during the data‐collection period were excluded.

### 2.3. Sample Size

The sample size was estimated based on the empirical rule of thumb requiring at least 10 respondents per measurement item [51]. The survey comprised four sections totaling 38 items (8, 5, 16, and 9 items, respectively). The baseline required sample size was calculated as: *N* = (8 + 5 + 16 + 9) × 10 = 380. Assuming an anticipated 20% rate of invalid questionnaires or participant dropout, the sample size was further expanded as: *N* = 380 ÷ (1 − 20%) = 475. Therefore, a minimum of 475 valid participants was required to ensure adequate statistical power for this study.

### 2.4. Data Collection

Data were collected using an online questionnaire platform. The questionnaire, including all study measures, was programmed on the platform, and a shareable QR code was generated. The first page of the questionnaire provided detailed information on the study objectives, instructions for completing the questionnaire, and an electronic informed consent form. Before data collection, the research team contacted hospital administrators. With their assistance, the electronic questionnaire was distributed through WeChat. Participants could access the questionnaire only after providing electronic informed consent. All participants were informed of their right to withdraw from the study at any time without penalty. Participants who had questions while completing the questionnaire could contact the research team for clarification.

To enhance data quality, the questionnaire platform was configured to allow only one submission from each IP address, and all items were set as mandatory. A total of 800 eligible ICU nurses accessed and participated in the survey via a WeChat questionnaire link or QR code. Of these participants, 50 nurses withdrew from the survey, 36 responses were incomplete due to mid‐survey dropout, and an additional 27 questionnaires were deemed invalid and excluded because all items contained identical responses, indicating an obvious pattern of abnormal responding. Ultimately, 687 valid questionnaires were retained, yielding an effective response rate of 85.88%.

### 2.5. Instruments

#### 2.5.1. Sociodemographic Characteristics

A self‐designed demographic questionnaire was used, including items on gender, age, marital status, educational level, working time in ICU (years), monthly income, having the thought to resign, and monthly night shifts.

#### 2.5.2. Surface Acting Scale

Surface acting was assessed using the surface acting subscale of the Emotional Labor Strategy Scale, originally developed by Grandey [52], and translated and adapted into Chinese by Wu Peijun. The subscale comprises five items, including “When interacting with service recipients (patients), I hide my true emotions to display appropriate emotions” and “Displaying proper emotions at work feels like acting to me.” Responses are rated on a five‐point Likert scale ranging from 1 (*strongly disagree*) to 5 (*strongly agree*). Total scores range from 5 to 25, and the mean item score ranges from 1 to 5. Higher scores indicate a greater tendency toward surface acting. In the present study, the Cronbach’s α coefficient was 0.861.

#### 2.5.3. Self‐Regulatory Fatigue Scale

Self‐regulatory fatigue was assessed using the Self‐Regulatory Fatigue Scale, developed by Nes et al. [53], and translated into Chinese by Ligang Wang. The scale comprises 16 items across three dimensions: cognitive fatigue (6 items), emotional fatigue (5 items), and behavioral fatigue (5 items). Items are rated on a five‐point Likert scale ranging from 1 (*strongly disagree*) to 5 (*strongly agree*). Total scores range from 16 to 80, with higher scores indicating greater self‐regulatory fatigue. The scale has been shown to distinguish self‐regulatory fatigue from deficits in self‐control and physical fatigue. In the present study, the Cronbach’s α coefficient was 0.842.

#### 2.5.4. Work Engagement Scale

Work engagement was assessed using the nine‐item Utrecht Work Engagement Scale (UWES‐9), originally developed by Schaufeli et al. [54] and translated and adapted into Chinese by Guangli Zhao. The scale comprises three dimensions: vigor (three items), dedication (three items), and absorption (three items). Items are rated on a seven‐point Likert scale ranging from 0 (*never*) to 6 (*every day*). Total scores range from 0 to 54, with higher scores indicating greater work engagement. In the present study, the Cronbach’s α coefficient was 0.965.

### 2.6. Data Analysis

First, IBM SPSS 26.0 was used to test for common method bias, perform descriptive statistics, and conduct correlation analyses. Continuous variables were described using means and standard deviations, and categorical variables were described using frequencies and percentages. All questionnaire items in this study were set as mandatory responses. There were no item‐level missing data among the 687 valid questionnaires included in the analysis. Consequently, all analyses were conducted using complete‐case data.

Second, Mplus 8.3 was used to perform LPA. We performed LPA using individual items of the Self‐Regulatory Fatigue Scale. This approach can capture differentiated characteristics among individuals with higher precision, is more suitable for interpreting individual response patterns and the properties of latent profiles, and exhibits greater sensitivity to subtle distinctions across profiles. Furthermore, to evaluate the robustness of latent classification results derived from different analytical levels, we conducted supplementary LPA with the three dimension scores (cognitive, emotional, and behavioral) as observed variables. However, the profiles displayed generally parallel low‐moderate‐high gradients across cognitive, emotional, and behavioral dimensions, which made it difficult to uncover distinct patterns of item‐level responses. For the three‐profile, four‐profile, and five‐profile solutions, the smallest subgroup contained only 32, 32, and 24 participants, accounting for 4.7%, 4.7%, and 3.5% of the total sample, respectively. This suggests poor classification validity and limited practical applicability. In addition, psychometric properties and measurement invariance tests of the Self‐Regulatory Fatigue Scale in the present study revealed unsatisfactory confirmatory factor analysis (CFA) model fit: Comparative Fit Index (CFI) = 0.710, Tucker–Lewis Index (TLI) = 0.656, root mean square error of approximation (RMSEA) = 0.125, and standardized root mean square residual (SRMR) = 0.196. Accordingly, the 16 items of the Self‐Regulatory Fatigue Scale were adopted as manifest variables to explore heterogeneity in individual response patterns corresponding to varied manifestations of self‐regulatory fatigue. The identified profiles represented distinct latent patterns of self‐regulatory fatigue among ICU nurses. We started with a one‐profile model and gradually increased the number of profiles until the maximum likelihood method failed to produce a unique solution.

Model selection was based on information criteria, including the Akaike Information Criterion (AIC), Bayesian information criterion (BIC), and sample‐size‐adjusted BIC (aBIC). Lower values of AIC, BIC, and aBIC indicate better model fit. The Lo–Mendell–Rubin adjusted likelihood ratio test (LMRT) and bootstrapped likelihood ratio test (BLRT) were applied to further evaluate and compare model adequacy. A significant p value (*p* < 0.05) suggests that the model with *n* profiles is superior to the model with (*n* − 1) profiles. The entropy value (ranging from 0 to 1) measures classification accuracy of profiles; an entropy value ≥ 0.80 indicates high accuracy of profile assignment [55]. Third, the determinants of different latent profiles of self‐regulatory fatigue among ICU nurses were examined. Based on the optimal latent profile model identified in the second step, univariable analyses were conducted on participants’ demographic characteristics. Chi‐square tests (for categorical variables) and analysis of variance (for continuous variables) were used to screen statistically significant indicators. Multicollinearity was assessed for variables with statistical significance in univariable analyses. Multinomial logistic regression analysis was performed to further explore factors associated with the formation of distinct latent profiles of self‐regulatory fatigue. Statistically significant demographic variables and the total scores of surface acting and work engagement (rather than subdimension scores) were incorporated into the logistic regression model. A significance level of *p* < 0.05 was adopted for both univariable and logistic regression analyses.

### 2.7. Ethical Approval

This study was conducted in accordance with the ethical standards of the Declaration of Helsinki [56]. All participants were informed prior to the survey that they were free to withdraw from the study at any time without any consequences. The data collected were used exclusively for this research, and all personal information was kept strictly confidential. The research protocol was approved by the Clinical Research Ethics Committee of Xi’an Weiyang Baoshihua Xinglongyuan Hospital (Approval No. XACHQ‐YJ‐25‐068).

## 3. Results

### 3.1. Common Method Bias Analysis

Harman’s single‐factor test was adopted to assess common method bias. Exploratory factor analysis was performed on all items of the three scales with unrotated factor extraction to retain factors with eigenvalues greater than 1. A critical threshold of 40% variance explained by the first extracted factor is commonly used to indicate substantial common method bias. Harman’s single‐factor test identified four factors with eigenvalues above 1. The first factor accounted for 26.67% of the total variance, which was below the 40% threshold, demonstrating no substantial common method bias in this study [57].

### 3.2. Participant Characteristics

A total of 800 ICU nurses were surveyed in this study, and 687 valid questionnaires were ultimately retained, yielding an effective response rate of 85.88%. The mean age of participants was 31.84 years (SD = 6.12), and the average working time in ICU was 9.80 years (SD = 6.34). Most participants were female (88.6%), married (69.1%), and had a bachelor’s degree (69.0%). Among participants, 47.7% had a monthly income 3001–6000 CNY, and 76.7% worked 6–7 night shifts monthly. Additional demographic characteristics are presented in Table 1.

**TABLE 1:** Characteristics of participants (*N* = 687).

Variable	Number	Proportion (%)
Gender		
Male	78	11.4
Female	609	88.6
Age (years)		
≤ 29	291	42.4
30–39	303	44.1
≥ 40	93	13.5
Education level		
College	165	24.0
Undergraduate	474	69.0
Postgraduate and above	48	7.0
Marital status		
Unmarried	212	30.9
Married	475	69.1
Working time in ICU (years)		
≤ 2	67	9.8
3–5	150	21.8
6–9	166	24.2
≥ 10	304	44.3
Having the thought to resign		
Yes	196	28.5
No	491	71.5
Monthly night shifts (*n*)		
≤ 5	61	8.9
6–7	527	76.7
≥ 8	99	14.4
Monthly income (CNY)		
≤ 3000	131	19.1
3001–6000	328	47.7
≥ 6001	228	33.2

### 3.3. Descriptive Statistics and Correlations

Self‐regulatory fatigue among nurses was negatively correlated with work engagement and positively correlated with surface acting. Descriptive statistics and correlations among self‐regulatory fatigue, surface acting, and work engagement in ICU nurses are presented in Table 2.

**TABLE 2:** Descriptive statistics and correlations between surface acting, self‐regulatory fatigue, and work engagement.

Measure	*M*	SD	1	2	3	4	5	6	7	8
1. Surface acting	18.79	3.62	1							
2. Self‐regulatory fatigue	48.93	8.87	0.255^∗∗^	1						
3. Cognition	19.92	3.42	0.156^∗∗^	0.686^∗∗^	1					
4. Emotion	14.25	4.45	0.269^∗∗^	0.826^∗∗^	0.221^∗∗^	1				
5. Behavior	14.75	3.19	0.167^∗∗^	0.896^∗∗^	0.526^∗∗^	0.666^∗∗^	1			
6. Work engagement	37.17	13.01	−0.191^∗∗^	−0.148^∗∗^	−0.088^∗^	−0.120^∗∗^	−0.150^∗∗^	1		
7. Vigor	12.52	4.48	−0.187^∗∗^	−0.131^∗∗^	−0.078^∗^	−0.103^∗∗^	−0.139^∗∗^	0.970^∗∗^	1	
8. Dedication	12.65	4.84	−0.207^∗∗^	−0.151^∗∗^	−0.092^∗^	−0.120^∗∗^	−0.153^∗∗^	0.956^∗∗^	0.903^∗∗^	1
9. Absorption	12.00	4.30	−0.150^∗∗^	−0.141^∗∗^	−0.082^∗^	−0.119^∗∗^	−0.138^∗∗^	0.940^∗∗^	0.878^∗∗^	0.827^∗∗^

^∗^ *p* < 0.05.

^∗∗^ *p* < 0.01.

### 3.4. LPA

The 16 items of the Self‐Regulatory Fatigue Scale were used as indicator variables, and latent profile models with 1–5 profiles were fitted. Model fitting indices, classification accuracy, and fitting differences are presented in Table 3. The three‐profile model was identified as the optimal LPA model, which demonstrated satisfactory model fit (AIC = 27530.750, BIC = 27839.884, aBIC = 27620.324). Both LMRT and BLRT *p* values were less than 0.001, suggesting statistically significant classification. The entropy value was 0.942, reflecting high classification accuracy. The smallest profile contained 103 participants, accounting for 15.0% of the total sample, which guaranteed stable results. Model selection was not based solely on fit indices or test results. Although the four‐profile and five‐profile models exhibited lower information criteria and higher entropy values, and produced significant BLRT test results compared with the LMRT, their smallest subgroups contained merely 13 and 14 participants, accounting for 2.0% and 2.1% of the total sample, respectively. Extremely small subgroups may lead to unstable parameter estimates and reduce the replicability of the model in other samples, indicating poor classification validity and limited practical value [58]. In contrast, the three‐profile model outperformed alternative models in the comprehensive comparison of goodness‐of‐fit, classification significance, accuracy, and stability, and was therefore selected as the final optimal model (Figure 1).

**TABLE 3:** Model fit indices of latent profile analysis.

Model	AIC	BIC	aBIC	Entropy	LMRT	BLRT	Category probability
One profile	31441.998	31586.532	31484.928	—	—	—	—
Two profile	28993.004	29215.089	29059.507	0.926	< 0.001	< 0.001	0.404/0.595
Three profile	27530.750	27839.884	27620.324	0.942	< 0.001	< 0.001	0.327/0.522/0.151
Four profile	26991.872	27368.055	27104.518	0.955	0.261	< 0.001	0.019/0.326/0.146/0.507
Five profile	26439.469	26892.702	26575.188	0.952	0.071	< 0.001	0.048/0.021/0.333/0.447/0.149

*Note:* aBIC: sample‐size‐adjusted Bayesian information criterion; LMRT: Lo–Mendell–Rubin likelihood ratio test.

Abbreviations: AIC, Akaike information criterion; BIC, Bayesian information criterion; BLRT, bootstrapped likelihood ratio test.

**FIGURE 1:**
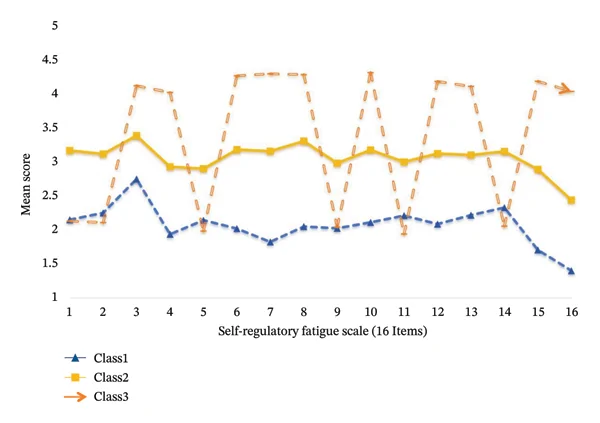
Latent profiles of self‐regulatory fatigue among ICU nurses. Note: Class 1: low self‐regulatory fatigue; Class 2: moderate self‐regulatory fatigue; Class 3: high self‐regulatory fatigue.

To further characterize the item‐level heterogeneity identified by the LPA, scores on the 16 items were compared across the three latent profiles: Class 1 (C1), Class 2 (C2), and Class 3 (C3) (Table 4). Several items showed the strongest differentiation across profiles, exhibiting a significant, monotonically increasing trend from the low self‐regulatory fatigue profile to the high self‐regulatory fatigue profile. These primarily included Item 9 (“I can handle stress well”), Item 15 (“I am always moody”), and Item 7 (“I am easily discouraged”) in the emotional dimension; Item 6 (“I always think about unpleasant things”) in the behavioral dimension; and Item 13 (“I find it difficult to remember things”) in the cognitive dimension. Furthermore, the item‐level analysis captured nonlinear patterns that were masked by dimension‐level sum scores. Specifically, for cognitive items such as Item 1 (“I feel full of energy”), Item 2 (“I can easily set goals”), and Item 11 (“It is very easy for me to stay in touch with friends”), the moderate self‐regulatory fatigue profile scored significantly lower than both the low and high self‐regulatory fatigue profiles. This highlights the unique cognitive and social coping heterogeneity among individuals during the self‐regulatory depletion process. To verify the reliability of the classification outcomes, average posterior classification probabilities of the three latent profiles were calculated as 98.1%, 97.3%, and 97.9% (Table 5). As shown in Table 4, scores on the self‐regulatory fatigue scale followed the order of Class 3 > Class 2 > Class 1 across the three latent subgroups. Combined with the characteristic analysis results of the latent profiles, Class 3, Class 2, and Class 1 were labeled as the high, moderate, and low self‐regulatory fatigue groups, respectively. The proportions of Class 1, Class 2, and Class 3 were 32.3%, 52.7%, and 15.0%, respectively.

**TABLE 4:** Comparison of self‐regulatory fatigue and item scores across latent profiles.

Item	C1: Low self‐regulatory fatigue (*n* = 222, 32.3%)	C2: Moderate self‐regulatory fatigue (*n* = 362, 52.7%)	C3: High self‐regulatory fatigue (*n* = 103, 15.0%)	*F*	*p*	LSD
Self‐regulatory fatigue	42.86 ± 5.58	48.11 ± 4.70	64.85 ± 6.71	607.205	< 0.001	C1 < C2 < C3
Cognition	20.46 ± 2.93	18.46 ± 2.68	23.86 ± 3.32	148.530	< 0.001	C2 < C1 < C3
1. I feel full of energy.	3.90 ± 0.87	2.91 ± 0.84	3.88 ± 0.88	113.935	< 0.001	C2 < C1 = C3
2. I can easily set goals.	3.79 ± 0.85	2.96 ± 0.78	3.91 ± 0.83	100.862	< 0.001	C2 < C1 = C3
3. I find it difficult to carry out my exercise plan.	2.69 ± 1.16	3.32 ± 0.84	4.02 ± 0.80	72.956	< 0.001	C1 < C2 < C3
5. Making decisions is not a problem for me.	3.90 ± 0.81	3.17 ± 0.70	4.04 ± 0.70	95.936	< 0.001	C2 < C1 = C3
11. It is very easy for me to stay in touch with friends.	4.02 ± 0.70	3.08 ± 0.68	3.98 ± 0.87	143.950	< 0.001	C2 < C1 = C3
13. I find it difficult to remember things.	2.17 ± 0.87	3.04 ± 0.76	4.03 ± 0.69	208.209	< 0.001	C1 < C2 < C3
Emotion	9.49 ± 2.45	15.31 ± 2.31	20.80 ± 2.21	901.239	< 0.001	C1 < C2 < C3
7. I am easily discouraged.	1.98 ± 0.71	3.11 ± 0.73	4.17 ± 0.49	388.546	< 0.001	C1 < C2 < C3
9. I can handle stress well.	1.79 ± 0.57	3.09 ± 0.75	4.20 ± 0.49	525.659	< 0.001	C1 < C2 < C3
12. I cry easily.	2.08 ± 0.88	3.10 ± 0.77	4.23 ± 0.53	286.211	< 0.001	C1 < C2 < C3
14. It is easy for me to maintain a healthy diet.	2.04 ± 0.90	3.06 ± 0.86	4.11 ± 0.70	225.031	< 0.001	C1 < C2 < C3
15. I am always moody.	1.68 ± 0.67	2.82 ± 0.80	4.12 ± 0.62	409.651	< 0.001	C1 < C2 < C3
Behavior	12.91 ± 2.18	14.33 ± 2.01	20.19 ± 2.32	431.796	< 0.001	C1 < C2 < C3
4. I have impulses to destroy something.	1.89 ± 0.98	2.88 ± 0.94	3.92 ± 0.94	170.338	< 0.001	C1 < C2 < C3
6. I always think about unpleasant things.	2.01 ± 0.78	3.23 ± 0.74	4.19 ± 0.53	366.358	< 0.001	C1 < C2 < C3
8. I try to avoid discussing or thinking about things that bother me.	3.84 ± 0.87	3.06 ± 0.78	4.09 ± 0.69	104.206	< 0.001	C2 < C1 < C3
10. I am always unable to control my temper.	3.72 ± 1.05	2.91 ± 0.80	3.98 ± 0.79	88.937	< 0.001	C2 < C1 < C3
16. I have impulses to hit or hurt someone.	1.38 ± 0.58	2.39 ± 0.98	3.97 ± 0.82	333.459	< 0.001	C1 < C2 < C3

*Note:* Data are presented as mean ± standard deviation. C1: low self‐regulatory fatigue; C2: moderate self‐regulatory fatigue; C3: high self‐regulatory fatigue. *p* values were obtained from one‐way analysis of variance. LSD indicates the results of post hoc least significant difference comparisons. “=” indicates no statistically significant difference between the two profiles (*p* > 0.05).

**TABLE 5:** Average latent profile probabilities for most likely latent class membership (row) by latent class (column).

Class	C1	C2	C3
C1	0.981	0.019	0.000
C2	0.020	0.973	0.007
C3	0.000	0.021	0.979

### 3.5. Characteristics of Latent Profile Membership

The results of the univariable analyses are presented in Table 6. Significant differences were observed among the three classes in gender, age, educational level, working time in ICU, having the thought to resign, monthly income, and monthly night shifts (all *p* < 0.05). The three latent classes also differed significantly in surface acting, work engagement, and their respective subdimensions (*p* < 0.05).

**TABLE 6:** Differences in demographic characteristics, surface acting, and work engagement across self‐regulatory fatigue latent profiles.

Variable	Respondents	C1: Low self‐regulatory fatigue (*n* = 222, 32.3%)	C2: Moderate self‐regulatory fatigue (*n* = 362, 52.7%)	C3: High self‐regulatory fatigue (*n* = 103, 15.0%)	*χ* ^2^/*F*	*p*
Gender					10.613	0.005
Male	78 (11.4%)	19 (8.6%)	54 (14.9%)	5 (4.9%)		
Female	609 (88.6%)	203 (91.4%)	308 (85.1%)	98 (95.1%)		
Age					55.546	< 0.001
≤ 29	291 (42.4%)	60 (27.0%)	176 (48.6%)	55 (53.4%)		
30–39	303 (44.1%)	105 (47.3%)	156 (43.1%)	42 (40.8%)		
≥ 40	93 (13.5%)	57 (25.7%)	30 (8.3%)	6 (5.8%)		
Education level					46.689	< 0.001
College	165 (24.0%)	36 (16.2%)	106 (29.3%)	23 (22.3%)		
Undergraduate	474 (69.0%)	151 (68.0%)	246 (68.0%)	77 (74.8%)		
Postgraduate and above	48 (7.0%)	35 (15.8%)	10 (2.8%)	3 (2.9%)		
Marital status					2.452	0.293
Unmarried	212 (30.9%)	61 (27.5%)	121 (33.4%)	30 (29.1%)		
Married	475 (69.1%)	161 (72.5%)	241 (66.6%)	73 (70.9%)		
Working time in ICU (years)					43.572	< 0.001
≤ 2	67 (9.8%)	17 (7.7%)	38 (10.5%)	12 (11.7%)		
3–5	150 (21.8%)	32 (14.4%)	93 (25.7%)	25 (24.3%)		
6–9	166 (24.2%)	35 (15.8%)	100 (27.6%)	31 (30.1%)		
≥ 10	304 (44.3%)	138 (62.2%)	131 (36.2%)	35 (34.0%)		
Having the thought to resign					34.546	< 0.001
Yes	196 (28.5%)	64 (28.8%)	79 (21.8%)	53 (51.5%)		
No	491 (71.5%)	158 (71.2%)	283 (78.2%)	50 (48.5%)		
Monthly night shifts (*n*)					56.990	< 0.001
≤ 5	61 (8.9%)	41 (18.5%)	14 (3.9%)	6 (5.8%)		
6–7	527 (76.7%)	163 (73.4%)	296 (81.8%)	68 (66.0%)		
≥ 8	99 (14.4%)	18 (8.1%)	52 (14.4%)	29 (28.2%)		
Monthly income (CNY)					12.790	0.012
≤ 3000	131 (19.1%)	56 (25.2%)	65 (18.0%)	10 (9.7%)		
3001–6000	328 (47.7%)	103 (46.4%)	169 (46.7%)	56 (54.4%)		
≥ 6001	228 (33.2%)	63 (28.4%)	128 (35.4%)	37 (35.9%)		
Surface acting	18.79 ± 3.62	17.87 ± 4.68	19.07 ± 2.99	19.76 ± 2.45	12.272	< 0.001
Work engagement	37.17 ± 13.01	39.21 ± 13.33	37.48 ± 12.46	31.68 ± 12.80	12.406	< 0.001
Absorption	12.00 ± 4.30	12.64 ± 4.43	12.09 ± 4.13	10.33 ± 4.20	10.558	< 0.001
Vigor	12.52 ± 4.48	13.19 ± 4.62	12.61 ± 4.23	10.71 ± 4.56	11.352	< 0.001
Dedication	12.65 ± 4.84	13.38 ± 4.99	12.78 ± 4.68	10.64 ± 4.55	11.920	< 0.001

### 3.6. Predictors of Latent Profile Membership

The multicollinearity diagnostic results showed that the variance inflation factor (VIF) values for the independent variables ranged from 1.048 to 6.216, whereas tolerance values ranged from 0.161 to 0.954. All values were within the acceptable ranges (VIF < 10, tolerance > 0.10) [59].

Variables that were statistically significant in the univariable analyses were entered into the multinomial logistic regression model, with Class 1 serving as the reference group (Table 7). Comparing Class 2 with Class 1: age ≤ 29 years, having the thought to resign, monthly night shifts ≥ 8, and higher surface acting scores predicted membership in Class 2, whereas age ≥ 40 years, postgraduate and above education level, unmarried marital status, monthly night shifts ≤ 5, monthly income ≤ 3000 CNY, and higher work engagement scores predicted membership in Class 1.

**TABLE 7:** Multinomial logistic regression analysis of predictors of latent profile membership among ICU nurses.

Variable	C2 vs. C1		95% CI	*p*	C3 vs. C1		95% CI	*p*
	*β*	OR			*β*	OR		
Gender (ref.: male)								
Female	−0.335	0.716	(0.241, 2.126)	0.547	0.639	1.894	(1.008, 3.560)	**0.047**
Age (ref.: 30–39)								
≤ 29	1.105	3.021	(1.040, 8.769)	**0.042**	0.558	1.747	(0.766, 3.986)	0.185
≥ 40	−1.032	0.356	(0.127, 0.999)	0.050	−0.784	0.457	(0.255, 0.819)	**0.009**
Education level (ref.: undergraduate)								
College	−0.039	0.962	(0.473, 1.954)	0.914	−0.563	0.570	(0.345, 0.940)	**0.028**
Postgraduate and above	−2.482	0.084	(0.020, 0.343)	**< 0.001**	−2.832	0.059	(0.024, 0.142)	**< 0.001**
Marital status (ref.: married)								
Unmarried	−1.144	0.318	(0.145, 0.699)	**0.004**	−0.681	0.506	(0.283, 0.905)	**0.022**
Working time in ICU (years) (ref.: ≥ 10)								
≤ 2	0.369	1.446	(0.321, 6.503)	0.631	0.584	1.793	(0.572, 5.625)	0.317
3–5	0.577	1.781	(0.483, 6.566)	0.386	0.940	2.559	(0.952, 6.880)	0.063
6–9	0.594	1.811	(0.742, 4.423)	0.192	0.828	2.289	(1.229, 4.263)	**0.009**
Having the thought to resign (ref.: no)								
Yes	0.985	2.679	(1.519, 4.724)	**< 0.001**	−0.449	0.638	(0.408, 0.999)	**0.049**
Monthly night shifts (*n*) (ref.: 6–7)								
≤ 5	−1.288	0.276	(0.099, 0.772)	**0.014**	−1.857	0.156	(0.078, 0.314)	**< 0.001**
≥ 8	1.397	4.042	(1.900, 8.599)	**< 0.001**	0.404	1.498	(0.792, 2.831)	0.214
Monthly income (CNY) (ref.: 3001–6000)								
≤ 3000	−1.189	0.305	(0.133, 0.700)	**0.005**	−0.286	0.751	(0.456, 1.237)	0.261
≥ 6001	0.011	1.011	(0.550, 1.857)	0.972	0.341	1.406	(0.895, 2.210)	0.139
Surface acting	0.141	1.151	(1.056, 1.255)	**< 0.001**	0.112	1.118	(1.059, 1.180)	**< 0.001**
Work engagement	−0.044	0.957	(0.936, 0.979)	**< 0.001**	−0.001	0.999	(0.984, 1.014)	0.911

*Note:* Bold values indicate *p* < 0.05.

Comparing Class 3 with Class 1: female gender, 6–9 years of working time in ICU, and higher surface acting scores predicted membership in Class 3, whereas age ≥ 40 years, college education or postgraduate and above education level, unmarried marital status, having the thought to resign, and monthly night shifts ≤ 5 predicted membership in Class 1.

## 4. Discussion

### 4.1. Latent Profile Patterns of Self‐Regulatory Fatigue Among ICU Nurses

This study employed LPA from a person‐centered perspective to explore the heterogeneity of self‐regulatory fatigue among ICU nurses. A key finding was the identification of three distinct latent profiles, providing empirical evidence for identifying high‐risk subgroups and developing targeted intervention strategies.

Specifically, three distinct profiles were identified: low self‐regulatory fatigue (32.3%), moderate self‐regulatory fatigue (52.7%), and high self‐regulatory fatigue (15.0%). This finding was consistent with previous studies [60, 61]. The moderate self‐regulatory fatigue profile accounted for the largest proportion, indicating that more than half of the ICU nurses experienced moderate levels of psychological resource depletion. Although the high self‐regulatory fatigue profile represented a small proportion of the total sample, the absolute number of affected nurses may still be considerable given the size of the overall nursing workforce. Moreover, this profile had significantly higher total scores on the Self‐Regulatory Fatigue Scale and its cognitive, emotional, and behavioral subdimensions than the other two groups. Nurses in this subgroup may experience considerable psychological resource depletion and face elevated risks of burnout, turnover intention, and nursing errors, suggesting that nurses with high self‐regulatory fatigue should not be overlooked.

This study identified significant heterogeneity among ICU nurses in total self‐regulatory fatigue scores and in the cognitive, emotional, and behavioral subdimensions, thereby supporting Hypothesis 1. According to the COR theory, self‐regulatory fatigue refers to the depletion of psychological resources resulting from sustained self‐control, emotion regulation, and behavioral restraint [62]. A meta‐analysis of 46 studies involving 39,246 ICU nurses indicated that ICU nurses frequently engage in high‐risk clinical decision‐making, care for critically ill patients, and experience substantial emotional strain and moral distress; emotional exhaustion is a critical predictor of turnover intention [63]. Their work environments are characterized by confined spaces, heavy workloads, a fast pace of work, and substantial clinical responsibilities. ICU nurses are responsible for providing patient care, participating in clinical decision‐making, and delivering palliative care when necessary. Simultaneously, they need to maintain professional, stable, and appropriate emotional expressions when interacting with patients, family members, physicians, and colleagues [64]. Persistent job demands may continuously deplete personal psychological resources, which in turn leads to varying degrees of cognitive, emotional, and behavioral exhaustion among nurses.

Notably, the distribution of cognitive fatigue across profiles differed significantly from that of emotional and behavioral fatigue, showing a pattern of Class 2 < Class 1 < Class 3 in the cognitive dimension. This finding suggests that self‐regulatory fatigue experienced by ICU nurses is not a simple linear accumulation process. Even when the overall fatigue level is low, nurses may still suffer from high cognitive fatigue. The cognitive dimension assesses individuals’ ability to regulate attention, process information, and think rationally. The emotional dimension reflects the capacity to manage emotions during prolonged self‐regulation, whereas the behavioral dimension measures the ability to sustain goal‐directed behaviors and inhibit impulses. ICU nursing involves complex tasks, including continuous patient monitoring, risk prediction, equipment parameter identification, medical order verification, emergency management, and multitask coordination [65]. For ICU nurses, unlike emotional regulatory fatigue, which mainly stems from emotional suppression and interpersonal interactions, cognitive fatigue is more closely linked to sustained attention, complex judgment, and a heavy clinical decision‐making burden, and is primarily manifested as a reduced sense of control over complicated work, daily life, and interpersonal communication. Nurses in Class 2 may experience obvious emotional exhaustion and behavioral regulatory burden, yet retain a certain sense of cognitive control over clear clinical protocols and routine work procedures, leading to relatively lower cognitive fatigue. By contrast, nurses in Class 1 had low overall fatigue but higher cognitive fatigue than those in Class 2. This indicates that some nurses encounter elevated cognitive load and a diminished sense of control amid continuous information processing, frequent task switching, and complex clinical situations.

### 4.2. Predictors of Latent Profiles of Self‐Regulatory Fatigue Among ICU Nurses

Statistically significant differences were found in the sociodemographic characteristics across subgroups, thereby supporting Hypothesis 2. Nurses with low self‐regulatory fatigue were more likely to be older, to have higher educational attainment, and to work fewer monthly night shifts compared with other profiles. Older nurses generally possess more accumulated clinical experience and may develop stable clinical judgment, emotion regulation skills, and problem‐solving strategies. When confronted with high‐stress situations, such as changes in critically ill patients’ conditions, emergency resuscitation, interdisciplinary collaboration, and nurse–patient communication, they may have a stronger sense of occupational control and a greater capacity to mobilize resources. This may reduce the depletion of resources related to cognitive control, emotion regulation, and behavioral control. Nurses with fewer monthly night shifts reported lower self‐regulatory fatigue, consistent with previous findings [66]. Reducing the number of night shifts may provide nurses with more opportunities for sleep, rest, and psychological recovery. Existing research indicates that fatigue induced by night shifts can be reversed after adequate recovery [67]. Fewer night shifts may result in less disruption of circadian rhythms and more sufficient recovery of sleep, physical functioning, and mental functioning, thereby stabilizing cognitive, emotional, and behavioral resources. Higher educational attainment may enhance nurses’ ability to integrate professional knowledge, engage in evidence‐based nursing practice, and solve complex clinical problems. These abilities may reduce feelings of uncertainty and powerlessness at work, minimize additional psychological resource depletion, and lower nurses’ levels of self‐regulatory fatigue.

Nurses in the moderate self‐regulatory fatigue profile were more likely to be aged ≤ 29 years, married, to work ≥ 8 monthly night shifts, and to report having the thought to resign. Younger nurses are generally at an early stage of career development. Although they possess fundamental clinical nursing competencies, their clinical experience and coping strategies may not be fully developed when facing intense work demands, making them more vulnerable to the depletion of self‐regulatory resources. Previous studies have also indicated that younger nurses are prone to psychological resource depletion under heavy workloads and complex clinical situations [68]. In addition to clinical duties, married nurses may shoulder multiple responsibilities, including family care, children’s education, and the coordination of domestic roles. Work–family role conflict may further exacerbate the depletion of their psychological resources [69]. Furthermore, frequent night shifts directly exacerbate sleep deprivation and circadian disruption, requiring nurses to expend extra self‐regulatory resources to maintain attention and emotional stability during physiologically vulnerable nighttime hours. A systematic review of qualitative studies involving 601 nurses from 11 countries found that night shifts disrupted circadian rhythms and impaired physical and mental health. Cumulative negative emotions may eventually develop into chronic fatigue [70].

Having the thought to resign may serve as an external manifestation of nurses’ work‐related stress, occupational burnout, or insufficient resources. Self‐regulatory fatigue may leave nurses feeling drained, struggling with emotion regulation, and experiencing a diminished sense of professional meaning at work, which may in turn trigger or reinforce the thought to resign. Persistent thoughts to resign and career uncertainty may further deplete psychological resources [71]. These findings suggest that self‐regulatory fatigue may follow a dynamic trajectory, with sustained stress and continuous resource depletion potentially shifting nurses from a low‐ to a high‐fatigue status over time.

The characteristics of the high self‐regulatory fatigue profile were mainly reflected in female gender, an ICU working time of 6–9 years. Female nurses were more likely than male nurses to belong to the high self‐regulatory fatigue profile. This may be attributed to the fact that female nurses often undertake higher levels of emotional labor and clinical empathy in ICU settings, while frequently managing the dual pressures of intense nursing duties and family responsibilities, thereby accelerating the depletion of their self‐regulatory resources [72]. Moreover, nurses with 6–9 years of ICU experience faced the highest risk of high self‐regulatory fatigue. Nurses at this stage typically serve as the core clinical backbone in ICUs, undertaking complex emergency resuscitations, managing the most critical patients, and bearing heavy clinical mentoring and administrative duties, leading to sustained mental and physical exhaustion [73].

This study further confirms that surface acting and work engagement are closely associated with self‐regulatory fatigue among ICU nurses, with significant differences across subgroups, thereby supporting Hypothesis 3. Goffman’s dramaturgical theory provides a framework for understanding the emotional displays of nursing staff during clinical interactions [74], whereas COR theory elucidates how the depletion of positive resources may contribute to fatigue patterns [75]. To maintain the emotional expressions required by their professional roles, ICU nurses may expend substantial psychological resources through surface acting, consequently exacerbating self‐regulatory fatigue. Self‐regulatory fatigue may deplete positive resources, such as vigor, dedication, and absorption, leading to reduced work engagement, whereas abundant positive resources may mitigate self‐regulatory fatigue. These two theories provide perspectives for understanding the relationships among professional role requirements, surface acting, resource status, and self‐regulatory fatigue among ICU nurses. Specifically, higher surface acting scores were associated with a greater likelihood of belonging to the moderate or high self‐regulatory fatigue profiles, which is consistent with findings from previous studies [76]. Goffman’s dramaturgical theory explains the motivations underlying surface acting among ICU nurses. This theory posits that individuals engage in impression management during social interactions in accordance with specific contexts and role expectations. Nurses regulate their emotions not merely due to personal preferences but to uphold a professional image, shaped jointly by professional norms, organizational expectations, and clinical interaction contexts. In practice, ICU nurses are required to display standardized professional emotions and maintain a steady professional demeanor when confronting anxious family members, critically ill patients, and communication conflicts. When nurses’ internal emotional states conflict with professional role expectations, they may conceal their genuine feelings and engage in surface acting. COR theory further explains how surface acting may contribute to self‐regulatory fatigue. According to this theory, tension, stress, and fatigue may emerge when resources are continuously depleted, threatened with loss, or invested without adequate replenishment. Although surface acting may facilitate smooth workplace interactions and preserve an organizational image in the short term, such impression management consumes substantial psychological resources and requires persistent suppression of inner emotions, as well as regulation of speech, facial expressions, and behaviors. This form of emotional labor may gradually erode self‐regulatory capacity and ultimately contribute to self‐regulatory fatigue. This also explains why nurses with higher levels of surface acting were more likely to belong to the moderate and high self‐regulatory fatigue classes.

Higher work engagement scores were associated with lower levels of self‐regulatory fatigue, highlighting its protective role, which aligns with previous research [77]. Work engagement encompasses three dimensions: vigor, dedication, and absorption, which reflect sustained energy, a strong sense of occupational meaning, and deep immersion in work. According to the COR theory, work engagement is not merely a positive psychological state but also a vital psychological resource [78]. Highly engaged nurses may derive a sense of achievement, value, and positive feedback from nursing practice, which may partially offset resource depletion triggered by heavy job demands. Specifically, vigor sustains adequate energy and recovery capacity amid high‐intensity work. Dedication fosters professional pride and a strong sense of purpose in nursing, thereby sustaining intrinsic motivation under stressful circumstances. Absorption promotes efficient task engagement and reduces depletion caused by distraction, hesitation, and negative mood. Therefore, higher work engagement may mitigate the risk of self‐regulatory fatigue by fostering positive emotional experiences, enhancing occupational control, and facilitating resource acquisition. Conversely, nurses with low work engagement may fail to derive sufficient meaning and positive rewards from their work, which may be accompanied by diminished professional identity and severe self‐regulatory fatigue. Notably, although univariable analyses showed differences in work engagement across the three latent profiles, after controlling for surface acting and other covariates, lower work engagement was associated only with membership in the moderate self‐regulatory fatigue profile (Class 2) and did not independently distinguish the high self‐regulatory fatigue profile (Class 3) from the low self‐regulatory fatigue profile (Class 1). This finding suggests that the apparent association between work engagement and high self‐regulatory fatigue may be partly influenced by surface acting and other related factors. In addition, the relatively small sample size of the high self‐regulatory fatigue profile (Class 3) may have reduced the statistical power to detect this association.

This study has several limitations. First, as a cross‐sectional study, it can only describe associations between variables and cannot infer causal relationships between self‐regulatory fatigue and its associated factors. Future studies should adopt longitudinal or experimental designs to further explore relevant causal pathways. Second, convenience sampling was used to recruit ICU nurses from tertiary hospitals in nine regions, which may have introduced selection bias and limited the representativeness and generalizability of the findings. Multicenter stratified random sampling designs could be adopted in future research to recruit nurses from diverse regions, hospitals at different levels, and various types of ICUs, thereby further verifying the stability and distribution of the latent profiles. Third, all data were collected through self‐administered questionnaires completed by nurses. Although Harman’s single‐factor test did not indicate serious common method bias, the findings may still have been affected by recall bias, social desirability bias, and common method bias. In particular, surface acting, work engagement, and self‐regulatory fatigue were all assessed through subjective self‐reports; therefore, the observed associations may have been overestimated to some extent. Future studies could employ longitudinal designs, multisource data (e.g., supervisor or colleague ratings), and objective measures, such as behavioral observations or physiological indicators, to further validate the relationships among these variables. Fourth, this study did not include key organizational‐level variables, such as staffing levels, leadership support, team climate, organizational justice, and managerial support, which may limit a comprehensive interpretation of the mechanisms underlying self‐regulatory fatigue among ICU nurses. Future research could incorporate ward‐level and hospital‐level indicators, including nurse‐to‐patient ratios, shift scheduling patterns, and departmental resources. A multilevel research design could be used to examine whether these organizational factors are associated with membership in the high self‐regulatory fatigue profile and to further identify modifiable organizational factors associated with self‐regulatory fatigue. Finally, the CFA of the three‐factor model of the Self‐Regulatory Fatigue Scale in this study did not meet the recommended fit criteria, suggesting that the scale may have limited factorial validity among ICU nurses. This finding may reflect genuine structural differences in the manifestation of self‐regulatory fatigue in ICU nurses compared with the original validation population; alternatively, it may indicate potential measurement issues with the scale in this group. Although item‐level LPA can more precisely capture individual response patterns and their heterogeneity, it cannot address the fundamental question of whether the scale’s three‐dimensional factor structure is applicable to ICU nurses. Future psychometric research in larger, multicenter samples of ICU nurses is needed to further validate the factor structure and measurement validity of this scale. Despite these limitations, this study provides valuable insights into the status of self‐regulatory fatigue among ICU nurses.

## 5. Conclusion

In summary, using LPA, we identified three distinct profiles of self‐regulatory fatigue among ICU nurses. Most ICU nurses exhibited moderate self‐regulatory fatigue, whereas smaller subgroups experienced high or low levels of self‐regulatory fatigue. In addition, we identified several factors associated with self‐regulatory fatigue classification among these ICU nurses. These predictors included gender, age, education level, marital status, ICU working time, having the thought to resign, monthly night shifts, monthly income, surface acting, and work engagement. The substantial individual differences among ICU nurses suggest that tailored interventions addressing the specific needs of each subgroup may maximize intervention effectiveness.

## 6. Implications for Nursing Management

This study identified three latent profiles of self‐regulatory fatigue among ICU nurses (low, moderate, and high) and examined factors associated with profile membership. The findings provide evidence for nursing managers to implement tiered and targeted interventions. For nurses in the high self‐regulatory fatigue profile, managers should pay close attention to female nurses, mid‐career nurses with substantial ICU experience, and those with heavy night‐shift workloads or prominent surface acting. Context‐specific interventions tailored to ICU practice are proposed. First, case discussions and scenario‐based simulations can help nurses identify high‐stress situations that may trigger emotional suppression or surface acting, such as unsuccessful resuscitation, sudden deterioration in patients’ conditions, communication conflicts with family members, and handovers during demanding night shifts. Second, nurses can receive training in emotion recognition, emotion labeling, cognitive reappraisal, and problem‐focused coping strategies. Third, role‐playing and communication‐feedback training can improve nurses’’ capacity to provide empathic responses, establish communication boundaries, and seek team support when interacting with anxious, angry, or grieving patients and their family members. Fourth, conducting structured emotional debriefing and providing peer‐led psychological support are recommended after high‐intensity incidents or resuscitation events to reduce the sustained accumulation of negative emotions. Beyond individual‐level training, organizational support is equally important. Nursing managers should allocate night‐shift staffing appropriately, minimize consecutive night shifts and demanding rotation schedules, and ensure adequate recovery time after night shifts. Appropriate job rotation, flexible rest arrangements, and psychological support services can be provided for nurses who undertake intensive emotional labor over long periods. In addition, career development guidance, career advancement opportunities, and work recognition mechanisms should be established to strengthen nurses’ sense of professional value and enhance work engagement.

For nurses in the moderate self‐regulatory fatigue profile, management priorities should focus on preventing progression to severe fatigue. ICU‐specific simulation training, professional competency development, and task‐management training may enhance their coping ability in high‐pressure clinical situations. A peer‐support system should be established in which experienced nurses provide clinical decision‐making guidance, emotional support, and practical mentoring. Managers can also organize stress management group activities and distribute work tasks reasonably to alleviate burdens arising from persistent emotional labor and behavioral self‐regulation.

For nurses in the low self‐regulatory fatigue profile, management strategies should focus on resource conservation and early prevention. Despite their overall low fatigue levels, they exhibited higher cognitive fatigue than nurses in the moderate self‐regulatory fatigue profile. This indicates that managers should continue to monitor their information‐processing workload, frequent task switching, pressure related to complex decision‐making, and work–life balance. Optimizing handover procedures, reducing unnecessary documentation burdens, providing clinical decision support tools, and offering opportunities for experience sharing and career development may help nurses maintain cognitive control and professional accomplishment while preventing progressive fatigue accumulation. Overall, nursing managers should adopt multilevel intervention strategies that integrate individual skill training, peer and managerial support, and optimized organizational work environments according to distinct self‐regulatory fatigue profiles and associated risk factors. Such strategies may reduce self‐regulatory fatigue among ICU nurses, enhance work engagement, and promote the stability and high‐quality development of nursing teams.

## Author Contributions

Hongli Zhang: conceptualization, methodology, validation, formal analysis, data curation, writing–original draft, and writing–review and editing. Yilin Wang and Mengyi Hu: validation and data curation. Yanting Han and Jiao Zhou: methodology and investigation. Qiaoying Yang: resources, project administration, and investigation. Ka Li: supervision, project administration, methodology, funding acquisition, conceptualization, and writing–review and editing.

## Funding

This study was supported by the Sichuan Provincial Department of Science and Technology (No. 2024YFFK0034).

## Disclosure

All authors have read and approved the final submitted version.

## Ethics Statement

This study was conducted in accordance with the ethical standards of the Declaration of Helsinki. All participants were fully informed prior to the survey that they were free to withdraw from the study at any time without any consequences. Their personal information was kept strictly confidential. The research protocol was approved by the Clinical Research Ethics Committee of Xi’an Weiyang Baoshihua Xinglongyuan Hospital (approval no.: XACHQ‐YJ‐25‐068).

## Conflicts of Interest

The authors declare no conflicts of interest.

## Supporting Information

Additional supporting information can be found online in the Supporting Information section.

## Supporting information


**Supporting Information** Results of the latent profile analysis for r dimensions and items of the Self‐Regulatory Fatigue Scale.

## Data Availability

The data that support the findings of this study are available upon request from the corresponding author. The data are not publicly available due to privacy or ethical restrictions.
