# Supplementary material for: Patterns and Predictors of Self‐Regulatory Fatigue Among Intensive Care Unit Nurses: A Person‐Centered Latent Profile Analysis
Source: J Nurs Manag. 2026 Oct 6;2026:4685274. doi: 10.1155/jonm/4685274 (PMC13641357; doi:10.1155/jonm/4685274)
Supplement: Supplementary file 1 [file JONM-2026-4685274-s001.docx]

1.Results of latent profile analysis using the three dimensions of self-regulatory fatigue (cognitive, emotional, and behavioral fatigue) as observed variables.

| Model fit indexes of latent profile analysis | | | | | | | |
| --- | --- | --- | --- | --- | --- | --- | --- |
| Model | AIC | BIC | aBIC | Entropy | LMR | BLRT | Category probability |
| One-profile | 11277.515 | 11304.709 | 11285.658 | — | — | — | — |
| Two-profile | 10687.689 | 10733.013 | 10701.261 | 0.827 | ＜0.001 | ＜0.001 | 0.316/0.684 |
| Three-profile | 10522.263 | 10585.716 | 10541.264 | 0.869 | ＜0.001 | ＜0.001 | 0.049/0.612/0.339 |
| Four-profile | 104133.485 | 10495.067 | 10437.914 | 0.818 | ＜0.001 | ＜0.001 | 0.483/0.292/0.048/0.177 |
| Five-profile | 10326.074 | 10425.786 | 10355.932 | 0.837 | 0.018 | ＜0.001 | 0.048/0.148/0.293/0.036/0.475 |
| Note: AIC: Akaike information criterion, BIC: Bayesian information criterion, ABIC: same-size adjusted Bayesian information criterion, LMR: Lo-Mendell-Rubin likelihood ratio test, VLMR: Vuong-Lo-Mendell-Rubin likelihood ratio test, BLRT: Bootstrapped likelihood ratio test | | | | | | | |

**2.Latent profile analysis results with the 16 items of the Self-Regulatory Fatigue Scale as manifest variables**

| Table 3 Model fit indexes of latent profile analysis (N = 687) | | | | | | | |
| --- | --- | --- | --- | --- | --- | --- | --- |
| Model | AIC | BIC | aBIC | Entropy | LMR | BLRT | Category probability |
| One-profile | 31441.498 | 31586.532 | 31484.928 | — | — | — | — |
| Two-profile | 28993.004 | 29215.089 | 29059.507 | 0.926 | ＜0.001 | ＜0.001 | 0.404/0.595 |
| Three-profile | 27530.750 | 27839.884 | 27620.324 | 0.942 | ＜0.001 | ＜0.001 | 0.327/0.522/0.151 |
| Four-profile | 26991.872 | 27368.055 | 27104.518 | 0.955 | 0.261 | ＜0.001 | 0.019/0.326/0.146/0.507 |
| Five-profile | 26439.469 | 26892.702 | 26575.188 | 0.952 | 0.071 | ＜0.001 | 0.048/0.021/0.333/0.447/0.149 |
| Note: AIC: Akaike information criterion, BIC: Bayesian information criterion, ABIC: same-size adjusted Bayesian information criterion, LMR: Lo-Mendell-Rubin likelihood ratio test, VLMR: Vuong-Lo-Mendell-Rubin likelihood ratio test, BLRT: Bootstrapped likelihood ratio test | | | | | | | |
